# Phone positioning influence in high-frequency audiometry

**DOI:** 10.1016/S1808-8694(15)31027-2

**Published:** 2015-10-19

**Authors:** Elizabeth Oliveira Crepaldi de Almeida, Aparecida Yumi Nishimori

**Affiliations:** a Audiology Specialist by the Federal Council of Speech and Hearing Therapy, CFFa; MsC in Communications Disorders - Pontifícia Universidade Católica de São Paulo, PUC-SP; PhD in Educational Psychology - Universidade Estadual de Campinas, UNICAMP. Full Professor of Speech and Hearing Therapy - PUC-Campinas.; b PIBIC-CNPQ scholarship holder, Undergraduate Student - Speech and Hearing Therapy Course - PUC-CAMPINAS. Pontifícia Universidade Católica de Campinas, PUC-Campinas.

**Keywords:** Audiometry, pure-tone, audiology, hearing, speech, language and hearing sciences

## Abstract

Research considers high frequency tonal audiometry as a tool for the early diagnosis of auditory alterations derived from etiological agents.

**Aim:**

to investigate possible differences in high frequency audiometry of individuals with normal hearing, based on the person who places the earphone.

**Patients and method:**

clinical and experimental study with 55 undergraduate students from a country side branch of the São Paulo State University, with normal hearing, underwent two tests each; for the first, the evaluator positioned the earphone on the participant; for the second one, the participant did it by him/herself. An AC40 audiometer calibrated to emit pure tone was used in the frequencies of 10, 12.5, and 16 kHz.

**Results:**

The kappa(k) coefficient statistical analysis was used to verify the agreement between the two ways of earphone positioning of earphone, bearing a ≥0.70 kappa value as a criterion. Results attained for both ears were below this criterion, with k average of 0.50.

**Discussion:**

results indicate a risk of compromising the exam reliability when the patient him/herself adjusts phone to his/her own ear.

**Conclusion:**

when performing audiometric assessment, this variable must be considered in order to attain reliable results.

## INTRODUCTION

High frequency audiometric threshold studies are important because the first indicators of a disease that affects the inner ear is a drop in the 8.000Hz^1^ frequency. This test helps in measuring, supporting and documentation of patients’ complaints about hearing acuity reduction, even when conventional audiometry is within normal ranges^2^.

Up to the 70's, this interest for audiologic evaluation focusing in the high frequencies was not so evident, having given the conclusions of other studies of the time that showed damages to the auditory system that could already be diagnosed early on by means of the conventional 8kHz frequency^2^, ^3^, ^4^, ^5^ assessment.

There are studies that have high frequencies tonal audiometry as a tool for the early diagnosis of hearing alterations caused by some etiological agents, such as aging, exposure to ototoxic agents, occupational noise, sequelae of otitis media, hearing monitoring in patients with hearing processing disorders, hearing impairment investigation in relatives of hearing impaired people of genetic origin^6^, ^7^, ^8^, ^9^, ^10^. Moreover, this type of audiometry may confirm the clinical impressions hinted by conventional audiometry^6^.

High frequency audiometry is a subjective hearing test, carried out in a soundproof booth, with earphones specially calibrated to emit extremely high sounds in the frequencies of 8,000 to 16,000Hz. There are audiometers able to generate pure tones of up to 20,000Hz, however there are no transducers able to emit tones above 16,000Hz, without distortions. It is considered an ideal exam to detect early phases of diseases that involve the auditory system and to monitor hearing risk status, such as cancer chemotherapy and the administration of potentially ototoxic drugs. Gender variability has been studied^12^, ^13^ and women have shown better hearing sensitivity when compared to men in the investigation of high frequency sounds. However, comparative studies count on the participation of a young population with normal hearing not presenting significant differences in results. There is no consensus in the literature in these regards, notwithstanding, it is known that women have more accurate hearing for intermediate and high frequencies^6^.

Considering hearing thresholds of up to 25dBHL (decibels Hearing Level) as normal hearing levels, we noticed an age-related progressive worsening in hearing acuity. Initially the impairment affects the frequency of 16kHz and, finally and with lesser intensity, the frequency of 10kHz^6^.

The difficulty in finding precise results led to the development of different assessment techniques. Notwithstanding, there are procedures that were created in order to solve this inter and intra-individual acoustic variability in the assessment of high frequencies that would not help in early diagnosis in clinical practice, when the assessments must rely on results and ease of application. For this reason, it is recommended to have special care in comparing findings in studies related to both normality and pathological state^8^.

It is important to inspect the external acoustic meatus in order to rule out the possibility of wax obstruction or foreign bodies. Moreover, errors may occur during the auditory evaluation by air conduction in the frequency of 8kHz, because the presence of a stationary wave may influence the sound intensity presented. If this is seen, we advice repositioning the phone, since there is a possibility for improvement in the threshold reached^16^.

This change in earphone positioning may alter the sound pressure level within the external acoustic meatus, varying according to sound frequency. In high frequencies there is some influence of the sound pressure level on the tympanic membrane, which also depends on the ear phone being well positioned^14^.

Earphone positioning may impact the assessment precision because when ¼ of the wavelength gets closer to the external meatus length, resonances and stationary waves are created, in such a way as to alter the initial test signal. This happens frequently with sounds above 3kHz. Above 15kHz this phenomena happens when half of the wavelength produces resonances in the cross-sections of the external auditory canal. There is the possibility of variations of about 15-20dB resulting from the slightest changes in earphone positioning in the ear^7^.

In clinical practice, it is possible to see a greater variability in tonal thresholds accruing from the collapse of the external acoustic meatus in the frequency of 4kHz, and most specially in the high frequencies^16^. In order to obtain a reliable result, phone adjustment or the use of an immittance probe may be of great worth in cases of meatus collapse, thus ruling out a “false” hearing alteration^4^. Moreover, the assessment becomes even more irregular in these cases, due to the fact that the tympanic membrane is positioned in an angle at the end of the external auditory meatus, and also there are anatomical variations in individuals that alter sound wave distribution^2^, ^3^, ^4^, ^14^.

The stationary wave pattern depends on the size of the outer ear, the middle ear impedance and sound source characteristics. A slight change in phone placement may also vary sound pressure on the tympanic membrane.

Due to test variabilities, the phones should be calibrated for each individual being tested, at each session and may be carried out by placing a probe microphone in the external acoustic meatus, thus allowing sound pressure measurement near the tympanic membrane^15^.

Thus, it is necessary to investigate the behavior of these thresholds in normal hearing individuals and test reliability, from detection to hearing impairment monitoring, specially in sensorineural pathologies, that usually start on the high frequencies^17^, ^18^.

## OBJECTIVE

Such study aims at checking if there are differences concerning the assessment of high frequencies in normal hearing individuals due to earphone positioning carried out by the investigator and the individual being tested.

This study is part of a greater project which investigates the audiologic characteristics of a group of students from an University in the country side of São Paulo State. In the present paper our goal is to compare the results of high frequency audiometric evaluation in normal hearing individuals with a change in ear phone position, when placed by the investigator and by the individual being tested.

It is expected that the evidence attained may offer valuable information for our field of work in regards to obtaining more precise diagnostic procedures, thus contributing for an early diagnosis and proper evaluation of the individuals tested.

## MATERIALS AND METHODS

Participants: 55 undergraduate students from an University in the country side of the State of São Paulo, with ages ranging between 18 and 25 years, with normal hearing.

## MATERIAL

We used an AC40 audiometer, calibrated to emit a pure tone at the specific frequency level, free from distortions and noise, thus assuring proper acoustic patterns in order to guarantee result reliability. The assessment was carried out up to the frequency of 16kHz because this is the frequency threshold of the equipment used. In order to analyze the results we used the SAS - System for Windows V8 - statistical package.

### Procedure

After the project was approved by the Research Ethics Committee (Protocol 185/05), we started collecting data. In order to select the participants in the study we carried out the conventional assessment involving patient interview, ear inspection, hearing threshold test, acoustic immittance and investigation of the stapedial reflexes. Only the participants with normal results in these tests were included in the high frequency audiometric assessment for this study. All the participants signed an informed consent form of their free will, according to Directive 196/96 from the Ministry of Health.

We surveyed the frequencies of 10; 12.5 and 16kHz, being the latter equal to the upper frequency limit of the equipment used. In order to investigate audiometric thresholds for conductive hearing, we used the descending technique with 10dB intervals in each frequency, until the individual no longer responded to the sound. As of such intensity, the ascending technique was used at 5dB intervals until the individual could hear the sound again. The hearing threshold corresponds to a lower sound intensity heard by the individual being assessed in each frequency.

Earphones were positioned by the investigators after briefly instructing the individuals on the task they should perform during the test. At the end of the threshold study in all the frequencies used, the individual herself removed and repositioned the earphone and a new threshold investigation was carried out in the frequencies of 10, 12.5 and 16kHz.

Results attained in the frequencies of 10, 12.5 and 16kHz, with earphone being positioned by the investigator were compared to the results obtained in the same frequencies after the individuals repositioned the phones themselves.

We controlled the variables related to test time and duration, room temperature, characteristics of that specific time of the day, and other variables that could harm results stability.

For result analysis, we used the Kappa (k) statistics, which is an agreement measure used in nominal scales and allows us to see how much the observations differ from expectations, based on chance, thus showing the legitimacy of such interpretations. k statistics values vary from 0 to 1; 0 indicates that there is no agreement except for sheer luck, and 1 represents perfect agreement^19^. In this case, since our goal is to assess whether or not the measure may be considered the same, regardless of who is placing the earphone on the subject being tested, we aim at achieving an agreement between the two ways of phone placing.

In order to obtain the k coefficient, we have to first calculate the representative index of agreement expected for the case. After that, we calculate the observed variable agreement. With these two values, we then calculate the k value by means of dividing the difference between the observed agreement and the expected one, by the difference between the absolute agreement and the expected agreement by chance (the largest possible difference between the observed and the expected agreement)^19^.

The k statistics magnitude is an agreement measure which is much more significant than its own statistical significance^19^. Results are interpreted in the following way, based on values taken by k: 0 indicates poor agreement; up to 0.20, slight agreement; from 0.21 to 0.40, considerable agreement; from 0.41 to 0.60, moderate agreement; from 0.61 to 0. 80, substantial agreement and from 0.80 to 1, excellent agreement^19^.

The k statistic standard error (SE) allows an estimate of its statistical significance and also its 95% confidence interval, calculated by the square root of the variable attained. Notwithstanding, the k value depends on the prevalence of the variable in question. A high prevalence results in a high level of agreement expected by chance, and this will result in a lower k value. On the other hand, a low prevalence variable will originate higher k values^19^.

For the present study we considered the Kappa coefficient above 0.70, substantial agreement, as a criterion. The confidence interval is of 95%, however the exact value of the estimate is between the minimum and maximum value in the range.

## RESULTS

Tables 1, 2, 3, 4, 5 and 6 show the crossed frequencies from the observations collected. In these tables, method # 1 represents the earphone placement by the investigator and method #2 is the placement carried out by the subject. The values presented by each method indicate the threshold obtained in decibels and the values in each cell represent the number of participants who answered at a given intensity. They also present the values obtained for the Kappa statistics, standard error and confidence interval (95%) for each frequency by both methods.

**Table 1:** Kappa statistics and analysis for the 10kHz frequency - right ear.

Crossed frequency from method 1 and method 2								
Method 1 Value in dB	Method 2 Value in dB							Total answers
	-10	-5	0	5	10	20	30	
-10	9	0	1	0	0	0	0	10
-5	2	8	4	1	0	0	0	15
0	0	1	5	3	1	0	0	10
5	0	3	2	6	1	0	0	12
10	0	0	1	0	2	1	0	4
15	0	0	1	0	0	1	0	2
20	0	0	0	0	0	1	0	1
30	0	0	0	0	0	0	1	1

Total answers	11	12	14	10	4	3	1	55
k	0.5053							
EP	0.0845							
Confidence Interval 95%		0.3396 to 0.6710

**Table 2:** Kappa statistics and analysis for the 12.5kHz - right ear.

Frequency from method 1 and method 2									
Method 1 Value in dB	Method 2 Value in dB								Total answers
	-10	-5	0	5	10	15	20	50	
-10	4	1	0	0	0	0	0	0	5
-5	3	9	4	0	0	0	0	0	16
0	0	4	10	1	0	0	0	0	15
5	0	0	1	3	2	0	0	0	6
10	0	0	1	2	2	0	0	0	5
15	0	0	1	1	0	4	0	0	6
20	0	0	0	0	0	0	1	0	1
50	0	0	0	0	0	0	0	1	1

Total answers	7	14	17	7	4	4	1	1	55
k	0.5233								
EP	0.0833								
Confidence Interval 95%		0.3600 to 0.6866

**Table 3:** Kappa statistics and analysis for the 16kHz - right ear.

Frequency from method 1 and method 2												
Method 1 Value in dB	Method 2 Value in dB											Total answers
	-10	-5	0	5	10	15	20	25	30	40	50	
-10	10	1	0	0	0	0	0	0	0	0	0	11
-5	1	5	3	1	0	0	0	0	0	0	0	10
0	1	3	4	3	1	0	0	0	0	0	0	12
5	0	0	2	5	0	1	0	0	0	0	0	8
10	0	1	0	0	2	0	0	0	0	0	0	3
15	0	0	0	0	0	1	1	0	0	0	0	2
20	0	0	0	0	0	1	3	1	0	0	0	5
35	0	0	0	0	0	0	0	0	0	1	0	1
50	0	0	0	0	0	0	0	0	1	0	2	3

Total answers	12	10	9	9	3	3	4	1	1	1	2	55
k	0.5392											
EP	0.0814											
Confidence Interval 95%		0.3796 to 0.6988

**Table 4:** Kappa statistics and analysis for the 10kHz frequency - left ear.

Frequency from method 1 and method 2									
Method 1 Value in dB	Method 2 Value in dB								Total answers
	-10	-5	0	5	10	15	25	50	
-10	5	2	0	0	0	0	0	0	7
-5	0	5	7	2	1	0	1	0	16
0	2	4	4	3	0	0	0	0	13
5	0	0	1	1	2	1	0	0	5
10	0	1	2	2	1	2	0	0	8
15	0	0	0	0	2	2	0	0	4
25	0	0	0	0	0	0	1	0	1
55	0	0	0	0	0	0	0	1	1

Total answers	7	12	14	8	6	5	2	1	55
k	0.2272								
EP	0.0822								
Confidence Interval 95%		0.0661 to 0.3883

**Table 5:** Kappa statistics and analysis for the 12.5kHz frequency - right ear.

Frequency from method 1 by method 2									
Method 1 Value in dB	Method 2 Value in dB								Total answers
	-10	-5	0	5	10	15	25	50	
-10	3	2	0	0	0	0	0	0	5
-5	5	9	2	1	0	0	0	0	17
0	0	5	7	1	0	0	0	0	13
5	0	0	1	4	2	0	0	0	7
10	0	0	1	0	4	0	0	0	5
15	0	0	0	0	3	2	0	0	5
20	0	0	0	0	0	0	0	1	1
30	0	0	0	0	0	0	1	0	1
50	0	0	0	0	0	0	0	1	1

Total answers	8	16	11	6	9	2	1	2	55
k	0.4597								
EP	0.0860								
Confidence Interval 95%		0.2912 to 0.6282

**Table 6:** Kappa statistics and analysis for the 16 kHz frequency - right ear.

Frequency from method 1 by method 2													
Method 1 Value in dB	Method 2 Value in dB												Total answers
											
-10	11	2	0	1	0	0	0	0	0	0	0	0	14
-5	1	3	1	0	0	0	0	0	0	0	0	0	5
0	0	1	3	2	2	0	0	0	0	0	0	0	8
5	0	1	3	1	0	0	0	0	0	0	0	0	5
10	0	0	0	3	1	0	1	0	0	0	0	0	5
15	0	0	0	0	2	2	1	0	0	0	0	0	5
20	0	0	0	0	0	1	0	1	0	0	0	1	3
25	0	0	0	0	0	0	1	1	0	0	0	0	2
30	0	0	0	0	0	0	1	0	0	0	0	0	1
35	0	0	0	0	0	0	0	0	0	1	0	0	1
40	0	0	0	0	0	0	0	0	0	0	1	0	1
50	0	0	0	0	0	0	0	0	3	0	0	2	5

Total answers	12	7	7	7	5	3	4	2	3	1	1	3	55
k	0.3992												
EP	0.0728												
Confidence Interval 95%:		0.2566 to 0.5419											

## DISCUSSION

Analyzing tables 1, 2 and 3, regarding right ear investigation under methods 1 and 2, in the frequencies of 10, 12.5 and 16kHz, we see that, although it bears a moderate agreement according to k values (0.5053; 0.5233; 0.5392) for the respective frequencies, they were below the criteria considered of 0.7 (substantial agreement). Thus, results attained in right ear studies show differences among the methods for earphone placement applied for audiometric threshold measurements.

Results analysis attained through the left ear analysis in the frequencies of 10, 12,5 and 16kHz, shown on tables 4, 5, and 6, also stressed a moderate agreement and, in the same way, below the criterion value of 0.7. It is worth to highlight that the k value in the 10kHz frequence was the lowest presented in this study.

For all the frequencies tested, we noticed in both right and left ears, differences in attaining auditory thresholds with the change in earphone positioning based on who placed the earphone, being either the examiner or the subject.

Such findings corroborate the results presented by Steffani et al.^20^, who showed the influence of earphone placement in order to obtain the hearing thresholds. In a similar fashion, Junqueira & Russo^16^ concluded that earphone adjustments and/or the immittance probe placement may enhance or worsen the exams.

According to Yonezaki^14^, earphone positioning has a significant influence during auditory threshold studies, specially in the frequencies of 6,000 and 8,000Hz, when the first results are considered altered.

Although these studies did not aim at assessing earphone positioning in high frequencies, they showed that phone positioning may impact audiologic assessment results. In a similar way, we observed in the present study that earphone placement by the examiner or by the examinee may impact high frequency audiologic assessment results.

## CONCLUSION

The agreement among the different measuring systems was considered to be low, with an average of 0.50. The measuring methods were considered different, because in all the cases the Kappa Statistical value was below 0.7, criterion deemed acceptable for this study. Even then, we may not state that there is no relation among the methods, because this average attained in the Kappa statistics is classified as moderate according to the literature^24^.

According to the analysis carried out, in all cases we noticed differences in hearing levels related to the person who placed the earphone. Thus, during an audiometric threshold investigation in the high frequencies, it is highly important to consider the interference of such variable when it is necessary to reposition the earphone in order to obtain reliable results.
